# Exploring Social Sustainability Through Urban Gardening: A Participatory Rapid Realist Review

**DOI:** 10.1093/geroni/igaf122.341

**Published:** 2025-12-31

**Authors:** Elise Stone, T J Roberts, Andrew Sixsmith, Mei Lan Fang

**Affiliations:** Simon Fraser University, Vancouver, British Columbia, Canada; Pendrellis Housing Society, Vancouver, British Columbia, Canada; Simon Fraser University, Vancouver, British Columbia, Canada; Simon Fraser University, Vancouver, British Columbia, Canada

## Abstract

Among the classic triad of sustainable development—ecology, economy, and society—social sustainability remains the least studied and critically evaluated. Conceptualizing wellbeing as a core component of social sustainability, this study explores how the social fabric of urban life, including third spaces, influences the wellbeing of older residents. Using a Participatory Rapid Realist Review (PRRR) methodology, co-designed with Pendrellis Housing Society, this research synthesizes peer-reviewed literature and integrates community-based perspectives to examine how urban gardening as a mechanism for developing engaging third places contributes to social sustainability. The PRRR approach investigates why, how, and under what circumstances urban gardening supports wellbeing. It follows a six-step process: (1) defining the review scope, (2) identifying relevant studies, (3) synthesizing data, (4) engaging stakeholders, (5) validating findings, and (6) disseminating results. Recognizing the complexity of linking urban gardening to wellbeing, this research takes a reflexive approach, embedding subjective perspectives within the analysis. Focusing on urban-dwelling older adults, gardening, and wellbeing, findings highlight the significance of small-scale social practices within third places that create vibrancy in urban life. Given the diversity of housing arrangements and motivations for gardening, understanding social sustainability at a micro scale is essential. Through community-engaged scholarship, older adults actively contributed to a co-designed Theory of Change, ensuring findings are grounded in lived experiences and applicable across the life course. Ultimately, this collaboration supported the development of an onsite community garden, ensuring older adults’ voices shaped both the research process and its practical application.

